# *N*-Acryloyl glycinamide

**DOI:** 10.1107/S1600536811029758

**Published:** 2011-07-30

**Authors:** Jan Seuring, Seema Agarwal, Klaus Harms

**Affiliations:** a Philipps Universität Marburg, Fachbereich Chemie, Hans-Meerwein-Strasse, D-35032 Marburg, Germany

## Abstract

The mol­ecule of the title compound [systematic name: *N*-(carbamoylmeth­yl)prop-2-enamide], C_5_H_8_N_2_O_2_, which can be radically polymerized to polymers with thermoresponsive behavior in aqueous solution, consists of linked essentially planar acryl­amide and amide segments [maximum deviations = 0.054 (1) and 0.009 (1) Å] with an angle of 81.36 (7)° between their mean planes. In the crystal, N—H⋯O hydrogen bonding leads to an infinite two-dimensional network along (100).

## Related literature

For the first preparation of the title compound, see: Haas & Schuler (1964 ▶). For the properties of polymers of the title compound in aquous solution, see: Haas *et al.* (1967 ▶, 1970**a* ▶,*b* ▶,*c* ▶,d* ▶); Marstokk *et al.* (1998 ▶); Nagaoka *et al.* (2007 ▶); Ohnishi *et al.* (2007 ▶); Seuring & Agarwal (2010 ▶); Glatzel *et al.* (2010 ▶). For the structure of the related compound, 2-(2-acryl­amido­acetamido)­acetic acid monohydrate, see Gao *et al.* (2007 ▶).
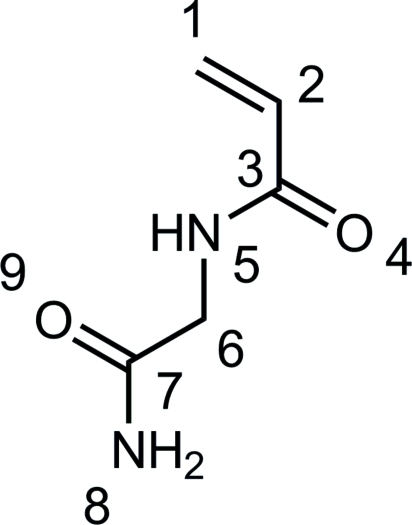


## Experimental

### 

#### Crystal data

C_5_H_8_N_2_O_2_
*M* *_r_* = 128.13
Monoclinic, 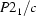

*a* = 15.938 (2) Å
*b* = 4.8055 (4) Å
*c* = 8.4920 (12) Å
β = 98.109 (11)°
*V* = 643.91 (14) Å^3^
*Z* = 4
Mo *K*α radiation
μ = 0.10 mm^−1^
*T* = 100 K
0.23 × 0.19 × 0.09 mm

#### Data collection

Stoe IPDS 2T diffractometer
Absorption correction: integration (*X-RED*; Stoe & Cie, 2006 ▶) *T*_min_ = 0.991, *T*_max_ = 0.997
6001 measured reflections
1362 independent reflections
1065 reflections with *I* > 2σ(*I*)
*R*_int_ = 0.049

#### Refinement

*R*[*F* ^2^ > 2σ(*F* ^2^)] = 0.032
*wR*(*F* ^2^) = 0.085
*S* = 0.97
1362 reflections
115 parameters
All H-atom parameters refined
Δρ_max_ = 0.18 e Å^−3^
Δρ_min_ = −0.15 e Å^−3^

### 

Data collection: *X-AREA* (Stoe & Cie, 2006 ▶); cell refinement: *X-AREA*; data reduction: *X-AREA*; program(s) used to solve structure: *SIR92* (Altomare *et al.*, 1994 ▶); program(s) used to refine structure: *SHELXL97* (Sheldrick, 2008 ▶); molecular graphics: *DIAMOND* (Brandenburg, 2007 ▶); software used to prepare material for publication: *publCIF* (Westrip, 2010 ▶), *PLATON* (Spek, 2009 ▶) and *WinGX* (Farrugia, 1999 ▶).

## Supplementary Material

Crystal structure: contains datablock(s) I, global. DOI: 10.1107/S1600536811029758/sj5179sup1.cif

Structure factors: contains datablock(s) I. DOI: 10.1107/S1600536811029758/sj5179Isup2.hkl

Supplementary material file. DOI: 10.1107/S1600536811029758/sj5179Isup3.cml

Additional supplementary materials:  crystallographic information; 3D view; checkCIF report

## Figures and Tables

**Table 1:** Hydrogen-bond geometry (Å, °)

*D*—H⋯*A*	*D*—H	H⋯*A*	*D*⋯*A*	*D*—H⋯*A*
N5—H5⋯O4^i^	0.850 (19)	2.062 (19)	2.8946 (14)	166.3 (15)
N8—H8*B*⋯O9^ii^	0.881 (17)	2.081 (17)	2.9494 (14)	168.2 (15)
N8—H8*A*⋯O9^iii^	0.927 (18)	1.971 (18)	2.8855 (14)	168.6 (16)

Symmetry codes: (i) 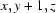; (ii) 
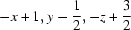
; (iii) 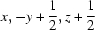.

**Fig. 1.**
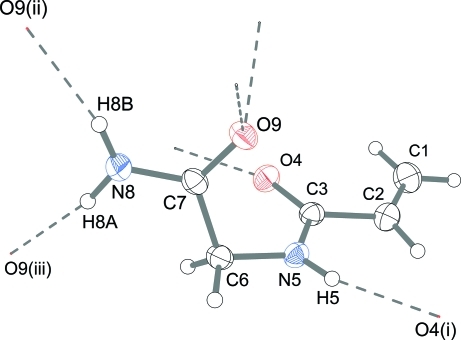
View of the crystal structure of the title compund. The thermal ellipsoids are drawn at 50% probability level. Dashed lines indicate hydrogen bonding contacts.

**Fig. 2.**
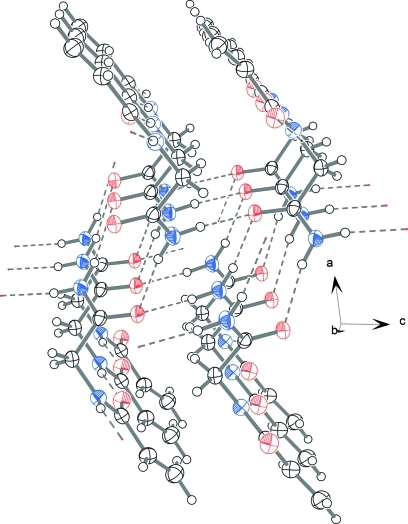
Two dimensional hydrogen bond network with plane direction (1 0 0). Dashed lines indicate hydrogen bonds.

## supplementary crystallographic information

### Comment

N-acryloyl glycinamide was first synthesized by Haas and Schuler (1964). It can be polymerized radically to obtain polymers that exhibit thermoresponsive behavior in aqueous solution. The polymers show gelatin-like thermoreversible gelation (Haas & Schuler, 1964; Haas *et al.*, 1967, 1970*a*, 1970*b*, 1970*c*, 1970*d*; Marstokk *et al.*, 1998; Seuring & Agarwal, 2010; Glatzel *et al.*, 2010) and an upper critical solution temperature (Seuring & Agarwal, 2010; Ohnishi *et al.*, 2007; Nagaoka *et al.*, 2007) in water. These phenomena rely on intermolecular hydrogen bonding. Therefore, investigating the intermolecular hydrogen bonding between monomer units in the crystal may contribute to the understanding of interpolymer interactions.

The molecular structure of the title compound shows two planar parts with C1, C2, C3, O4, N5, C6 and C6, C7, N8, O9 in plane. The angle between these mean planes is 81.36 (7)°. In the packing the molecule forms three hydrogen bonds to three different neighbouring molecules. For details see Table 1. The intermolecular N5—H5···O4^i^ contacts form an infinite chain in the (0 1 0) direction. Two of these chains are linked *via* N8—H8*A*···O9^iii^ and N8—H8*B*···O9^ii^ interactions, respectively (symmetry codes: (i) *x*, *y* + 1, *z*; (ii) -*x* + 1, *y* - 1/2, -*z* + 3/2; (iii) *x*, -*y* + 1/2, *z* + 1/2). Herein the N8—H8*A*···O9^iii^ hydrogen bonds form a second chain with direction (0 0 1), and hydrogen bonded rings are generated. In conclusion, a two dimensional hydrogen bond network has been formed with the hydrophobic tails of the molecules as border planes.

For the crystal structure of a related compound, 2-(2-acrylamidoacetamido)acetic acid monohydrate, see Gao *et al.* (2007).

### Experimental

N-acryloyl glycinamide has been prepared according to the route of Haas and Schuler (1964). However, reagent ratios, workup and purification have been modified as follows.

In a 1 l three-necked round-bottom flask equipped with a mechanical stirrer glycinamide hydrochloride (23.11 g, 209 mmol) and potassium carbonate (56.7 g, 410 mmol) were dissolved in 125 ml of water. The solution was cooled in an ice bath and acryloyl chloride (16.65 ml, 205 mmol) dissolved in 250 ml of diethylether was added dropwise over 30 min with fast stirring (300 rpm). The suspension was further stirred at RT for 2 h. The diethylether was removed by rotary evaporation at 35 °C. The remaining aqueous phase was freeze dried. The crude brittle solid was extracted with acetone (6 times, 500 ml acetone, 40 °C, stirring for at least 15 min). Insoluble potassium salts were filtered off and the acetone was removed by rotary evaporation at 35 °C. 22.7 g (85%) of crude product were obtained. The crude product was dissolved in an eluent mixture of methanol and dichloromethane (*v*/*v* = 1/4, 600 ml) by heating to reflux once. The solution was filtered to remove polymeric impurities and purified by column chromatography (d = 9 cm, 900 g silica, porosity 60 Å, 0.063–0.2 mm mesh size, TLC: *R*_f_(N-acryloyl glycinamide) = 0.40) to obtain 21.3 g (80%) of product which was recrystallized from 240 ml of a mixture of methanol and acetone (*v*/*v* = 1/2) to yield 15.3 g (57%) of colorless needle-like crystals.

For obtaining crystals that are suitable for X-ray analysis a small fraction of purified N-acryloyl glycinamide was again recrystallized from a dilute solution in 2-propanol.

### Refinement

All H atoms were located in a difference Fourier map and refined isotropically.  The C—H bond distances vary from 0.937 (18) to 0.981 (18), the N—H bond lenghts from 0.850 (19) to 0.927 (18) Å.

### Crystal data

**Table:**

C_5_H_8_N_2_O_2_	*F*(000) = 272
*M**_r_* = 128.13	*D*_x_ = 1.322 Mg m^−^^3^
Monoclinic, *P*2_1_/*c*	Melting point: 143 K
Hall symbol: -P 2ybc	Mo *K*α radiation, λ = 0.71073 Å
*a* = 15.938 (2) Å	Cell parameters from 7769 reflections
*b* = 4.8055 (4) Å	θ = 2.6–27°
*c* = 8.4920 (12) Å	µ = 0.10 mm^−^^1^
β = 98.109 (11)°	*T* = 100 K
*V* = 643.91 (14) Å^3^	Plate, colourless
*Z* = 4	0.23 × 0.19 × 0.09 mm

### Data collection

**Table:**

Stoe IPDS 2T diffractometer	1362 independent reflections
Radiation source: fine-focus sealed tube	1065 reflections with *I* > 2σ(*I*)
graphite	*R*_int_ = 0.049
Detector resolution: 6.67 pixels mm^-1^	θ_max_ = 26.8°, θ_min_ = 2.6°
rotation method scans	*h* = −20→17
Absorption correction: integration (*X-RED*; Stoe & Cie, 2006)	*k* = −6→6
*T*_min_ = 0.991, *T*_max_ = 0.997	*l* = −10→10
6001 measured reflections	

### Refinement

**Table:**

Refinement on *F*^2^	Secondary atom site location: difference Fourier map
Least-squares matrix: full	Hydrogen site location: difference Fourier map
*R*[*F*^2^ > 2σ(*F*^2^)] = 0.032	All H-atom parameters refined
*wR*(*F*^2^) = 0.085	*w* = 1/[σ^2^(*F*_o_^2^) + (0.0548*P*)^2^] where *P* = (*F*_o_^2^ + 2*F*_c_^2^)/3
*S* = 0.97	(Δ/σ)_max_ < 0.001
1362 reflections	Δρ_max_ = 0.18 e Å^−^^3^
115 parameters	Δρ_min_ = −0.15 e Å^−^^3^
0 restraints	Extinction correction: *SHELXL97* (Sheldrick, 2008), Fc^*^=kFc[1+0.001xFc^2^λ^3^/sin(2θ)]^-1/4^
Primary atom site location: structure-invariant direct methods	Extinction coefficient: 0.032 (7)

### Special details

**Table:**

Experimental. DSC (rate of heating = 10 K min^-1^): T_m_ = 143 °C. IR (ATR): ν= 3380 (m, NH), 3312 (s, NH), 3187 (m, NH), 1652 (*vs*, C=O), 1621 (*vs*, C=O), 1551 (*vs*, NH) cm^-1. 1^H NMR (300 MHz, D_2_O): δ = 3.93 (s, 2H, N–CH_2_–CONH_2_), 5.77 [dd, J(doublet 1) = 2.0 Hz, J(doublet 2) = 9.5 Hz, 1H, H_olef._], 6.20 [dd, J(doublet 1) = 2.0 Hz, J(doublet 2) = 17.1 Hz, 1H, H_olef._], 6.29 [dd, J(doublet 1) = 9.5 Hz, J(doublet 2) = 17.2 Hz, 1H, H_olef._]. ^13^C NMR (75 MHz, D_2_O): δ = 42.7 (–N—CH_2_–), 128.8 (C_olef._), 130.0 (C_olef._), 169.6 (–CO–), 174.8 (CO–). Flame emission spectroscopy: potassium content 5 p.p.m.
Geometry. All s.u.'s (except the s.u. in the dihedral angle between two l.s. planes) are estimated using the full covariance matrix. The cell s.u.'s are taken into account individually in the estimation of s.u.'s in distances, angles and torsion angles; correlations between s.u.'s in cell parameters are only used when they are defined by crystal symmetry. An approximate (isotropic) treatment of cell s.u.'s is used for estimating s.u.'s involving l.s. planes.
Refinement. Refinement of *F*^2^ against ALL reflections. The weighted *R*-factor *wR* and goodness of fit *S* are based on *F*^2^, conventional *R*-factors *R* are based on *F*, with *F* set to zero for negative *F*^2^. The threshold expression of *F*^2^ > 2σ(*F*^2^) is used only for calculating *R*-factors(gt) *etc*. and is not relevant to the choice of reflections for refinement. *R*-factors based on *F*^2^ are statistically about twice as large as those based on *F*, and *R*- factors based on ALL data will be even larger.

### Fractional atomic coordinates and isotropic or equivalent isotropic displacement parameters (Å^2^)

**Table:**

	*x*	*y*	*z*	*U*_iso_*/*U*_eq_	
C1	0.91738 (9)	0.4644 (3)	0.66968 (16)	0.0334 (3)	
C2	0.87077 (8)	0.6065 (3)	0.75755 (15)	0.0269 (3)	
C3	0.80276 (7)	0.4732 (3)	0.83438 (13)	0.0218 (3)	
C6	0.68733 (7)	0.5421 (3)	0.98560 (13)	0.0229 (3)	
C7	0.61296 (7)	0.4355 (3)	0.86952 (13)	0.0209 (3)	
N5	0.75513 (6)	0.6473 (2)	0.90665 (12)	0.0221 (3)	
N8	0.56460 (7)	0.2477 (2)	0.92691 (12)	0.0271 (3)	
O4	0.79110 (6)	0.21891 (18)	0.83202 (10)	0.0280 (2)	
O9	0.59828 (5)	0.52628 (19)	0.73164 (9)	0.0248 (2)	
H1A	0.9069 (10)	0.273 (4)	0.6562 (19)	0.038 (4)*	
H1B	0.9623 (10)	0.553 (4)	0.620 (2)	0.041 (4)*	
H2	0.8770 (10)	0.802 (4)	0.7774 (18)	0.036 (4)*	
H5	0.7628 (10)	0.822 (4)	0.8996 (19)	0.034 (4)*	
H6A	0.7081 (8)	0.395 (3)	1.0551 (17)	0.023 (3)*	
H6B	0.6661 (9)	0.697 (3)	1.0471 (17)	0.025 (3)*	
H8A	0.5799 (10)	0.181 (4)	1.029 (2)	0.042 (4)*	
H8B	0.5176 (11)	0.194 (4)	0.867 (2)	0.040 (4)*	

### Atomic displacement parameters (Å^2^)

**Table:**

	*U*^11^	*U*^22^	*U*^33^	*U*^12^	*U*^13^	*U*^23^
C1	0.0316 (7)	0.0359 (8)	0.0336 (7)	0.0022 (6)	0.0078 (5)	0.0017 (6)
C2	0.0260 (6)	0.0247 (7)	0.0300 (6)	−0.0003 (5)	0.0039 (5)	0.0015 (5)
C3	0.0246 (6)	0.0198 (6)	0.0201 (5)	0.0000 (5)	0.0001 (4)	0.0001 (4)
C6	0.0261 (6)	0.0237 (6)	0.0189 (6)	−0.0002 (5)	0.0028 (4)	−0.0010 (5)
C7	0.0231 (6)	0.0211 (6)	0.0193 (5)	0.0030 (5)	0.0058 (4)	−0.0008 (4)
N5	0.0244 (5)	0.0177 (6)	0.0246 (5)	−0.0012 (4)	0.0040 (4)	−0.0010 (4)
N8	0.0286 (5)	0.0318 (7)	0.0204 (5)	−0.0076 (4)	0.0020 (4)	0.0030 (4)
O4	0.0361 (5)	0.0180 (5)	0.0304 (5)	−0.0008 (4)	0.0065 (4)	−0.0011 (4)
O9	0.0273 (4)	0.0291 (5)	0.0178 (4)	0.0006 (4)	0.0028 (3)	0.0025 (3)

### Geometric parameters (Å, °)

**Table:**

C1—C2	1.3160 (19)	C6—C7	1.5198 (16)
C1—H1A	0.937 (18)	C6—H6A	0.950 (15)
C1—H1B	0.981 (18)	C6—H6B	0.994 (15)
C2—C3	1.4867 (17)	C7—O9	1.2405 (13)
C2—H2	0.956 (18)	C7—N8	1.3235 (16)
C3—O4	1.2360 (15)	N5—H5	0.850 (19)
C3—N5	1.3356 (16)	N8—H8A	0.927 (18)
C6—N5	1.4412 (15)	N8—H8B	0.881 (17)
			
C2—C1—H1A	118.2 (10)	N5—C6—H6B	108.4 (8)
C2—C1—H1B	121.6 (10)	C7—C6—H6B	107.4 (8)
H1A—C1—H1B	120.2 (15)	H6A—C6—H6B	110.1 (11)
C1—C2—C3	122.01 (13)	O9—C7—N8	123.05 (11)
C1—C2—H2	123.8 (10)	O9—C7—C6	121.30 (11)
C3—C2—H2	114.2 (10)	N8—C7—C6	115.62 (10)
O4—C3—N5	122.19 (11)	C3—N5—C6	120.42 (11)
O4—C3—C2	122.42 (11)	C3—N5—H5	119.2 (11)
N5—C3—C2	115.39 (11)	C6—N5—H5	120.2 (11)
N5—C6—C7	112.57 (9)	C7—N8—H8A	119.6 (11)
N5—C6—H6A	109.3 (8)	C7—N8—H8B	118.6 (11)
C7—C6—H6A	108.9 (8)	H8A—N8—H8B	121.8 (15)
			
C1—C2—C3—O4	6.58 (19)	O4—C3—N5—C6	−0.03 (16)
C1—C2—C3—N5	−173.59 (12)	C2—C3—N5—C6	−179.87 (10)
N5—C6—C7—O9	−26.19 (17)	C7—C6—N5—C3	−70.81 (14)
N5—C6—C7—N8	155.59 (11)		

### Hydrogen-bond geometry (Å, °)

**Table:**

*D*—H···*A*	*D*—H	H···*A*	*D*···*A*	*D*—H···*A*
N5—H5···O4^i^	0.850 (19)	2.062 (19)	2.8946 (14)	166.3 (15)
N8—H8B···O9^ii^	0.881 (17)	2.081 (17)	2.9494 (14)	168.2 (15)
N8—H8A···O9^iii^	0.927 (18)	1.971 (18)	2.8855 (14)	168.6 (16)

Symmetry codes: (i) *x*, *y*+1, *z*; (ii) −*x*+1, *y*−1/2, −*z*+3/2; (iii) *x*, −*y*+1/2, *z*+1/2.

## References

[bb1] Altomare, A., Cascarano, G., Giacovazzo, C., Guagliardi, A., Burla, M. C., Polidori, G. & Camalli, M. (1994). *J. Appl. Cryst.* **27**, 435.

[bb2] Brandenburg, K. (2007). *DIAMOND* Crystal Impact GbR, Bonn, Germany.

[bb3] Farrugia, L. J. (1999). *J. Appl. Cryst.* **32**, 837–838.

[bb4] Gao, X., Wu, C., Wang, H. & Wang, J. (2007). *Acta Cryst.* E**63**, o4580.

[bb5] Glatzel, S., Badi, N., Päch, M., Laschewsky, A. & Lutz, J.-F. (2010). *Chem. Commun.* **46**, 4517–4519. 10.1039/c0cc00038h 20485757

[bb6] Haas, H. C., Chiklis, C. K. & Moreau, R. D. (1970*a*). *J. Polym. Sci. Part A Polym. Chem.* **8**, 1131–1145.

[bb7] Haas, H. C., MacDonald, R. L. & Schuler, A. N. (1970*b*). *J. Polym. Sci. Part A Polym. Chem.* **8**, 3405–3415.

[bb8] Haas, H. C., MacDonald, R. L. & Schuler, A. N. (1970*c*). *J. Polym. Sci. Part A Polym. Chem*, **8**, 1213–1226.

[bb9] Haas, H. C., Manning, M. J. & Mach, M. H. (1970*d*). *J. Polym. Sci. Part A Polym. Chem.* **8**, 1725–1730.

[bb10] Haas, H. C., Moreau, R. D. & Schuler, N. W. (1967). *J. Polym. Sci. Part B Polym. Phys.* **5**, 915–927.

[bb11] Haas, H. C. & Schuler, N. W. (1964). *J. Polym. Sci. Part B Polym. Lett.* **2**, 1095–1096.

[bb12] Marstokk, O. B., Nyström, B. & Roots, J. (1998). *Macromolecules*, **31**, 4205–4212.

[bb13] Nagaoka, H., Ohnishi, N. & Eguchi, M. (2007). US Patent No. 0203313 A1.

[bb14] Ohnishi, N., Furukawa, H., Kataoka, K. & Ueno, K. (2007). US Patent No. 7,195,925 B2.

[bb15] Seuring, J. & Agarwal, S. (2010). *Macromol. Chem. Phys.* **211**, 2109–2117.

[bb16] Sheldrick, G. M. (2008). *Acta Cryst.* A**64**, 112–122. 10.1107/S0108767307043930 18156677

[bb17] Spek, A. L. (2009). *Acta Cryst.* D**65**, 148–155. 10.1107/S090744490804362X PMC2631630 19171970

[bb18] Stoe & Cie (2006). *X-AREA and *X-RED32** Stoe & Cie, Darmstadt, Germany.

[bb19] Westrip, S. P. (2010). *J. Appl. Cryst.* **43**, 920–925.

